# Small pancreatic ductal adenocarcinoma (≤ 2 cm): different imaging and clinicopathologic features according to extrapancreatic extension

**DOI:** 10.1007/s00261-025-04831-0

**Published:** 2025-02-14

**Authors:** Rae Rim Ryu, Jung Hoon Kim, Junghoan Park, Sungjun Hwang

**Affiliations:** 1 Chung-Ang University Hospital, Seoul, Korea, Republic of; 2 Seoul National University, Seoul, Korea, Republic of; 3 Seoul National University Hospital, Seoul, Korea, Republic of; 4 Inje University Ilsan Paik Hospital, Goyang-si, Korea, Republic of

**Keywords:** Pancreas, Neoplasm staging, Tomography, Magnetic resonance imaging

## Abstract

**Purpose:**

To assess features of small pancreatic ductal adenocarcinoma (s-PDA, ≤ 2 cm) according to extrapancreatic extension (EPE) and predictors for recurrence.

**Methods:**

This retrospective study included patients diagnosed with s-PDA who underwent surgery between January 2004 and October 2021. Preoperative CT or MRI images were reviewed by two reviewers. Imaging and clinicopathologic features of s-PDA were compared according to the presence of EPE. Cox regression analyses were performed to identify predictors of recurrence.

**Results:**

142 patients (77 men; 64.7 ± 9.3 years) who underwent preoperative CT (*n* = 134) or MRI (*n* = 115) were included. Duct dilatation was a common imaging finding of s-PDA (CT: 75.4%, MRI: 82.6%). Of the 142 patients, 21.8% (31/142) had no EPE, while 78.2% (111/142) had EPE. Tumor size on CT (14.3 ± 8.7 mm vs. 18.2 ± 6.5 mm, *p* =.01) and abutment or encasement of superior mesenteric vein (13.8% vs. 40.9%, *p* =.02) on CT were different according to absence or presence of EPE. Recurrence was more common in s-PDA with EPE (32.3% [10/31] vs. 53.2% [59/111], *p* =.04). Pathologic tumor size (HR 1.103, 95% CI 1.020–1.193, *p* =.01), tumor size on MRI (HR 1.044, 95% CI 1.001–1.090, *p* =.048), and extrapancreatic neural invasion on MRI (HR 3.341, 95% CI 1.564–7.140, *p* =.002) were significant predictors of recurrence.

**Conclusion:**

Even in s-PDA, tumors with EPE are larger and show higher recurrence rates. Imaging features are important for predicting presence of EPE.

**Supplementary Information:**

The online version contains supplementary material available at 10.1007/s00261-025-04831-0.

## Introduction

Pancreatic ductal adenocarcinoma (PDA) is the fourth leading cause of cancer-related mortality worldwide, with a 5-year survival rate of only approximately 12% [1, 2]. Due to late detection, only 10–20% of PDAs are operable at diagnosis [3], making early diagnosis crucial because surgery is the only curative treatment.

Typically, pancreatic cancer appears as a hypoattenuating mass on CT [4]. However, small PDAs (s-PDA), measuring 2 cm or less, often appear as isoattenuating masses, detectable only through secondary signs such as pancreatic duct cut-off, dilatation of the pancreatic duct or common bile duct (CBD), parenchymal atrophy, and contour abnormality [5, 6]. Despite advances in medical imaging, the diagnosis of s-PDA remains challenging.

Meanwhile, with the revision to the American Joint Committee on Cancer (AJCC) 8th Edition staging system, pancreatic cancer staging now depends solely on tumor size for T staging, and excludes the concept of extrapancreatic extension (EPE). The College of American Pathologists’ protocol defines extrapancreatic extension as the invasion of peripancreatic soft tissues, the extrapancreatic biliary system, the ampulla of Vater, or the duodenum. It further specifies that peripancreatic soft tissue includes the mesenteric fat, mesocolon, greater and lesser omentum, and the peritoneum [7]. T1 staging includes tumors equal to or less than 2 cm, regardless of EPE, leading to an increased prevalence of T1 cancers [8]. This change was based on a previous study that argued that peripancreatic soft tissue involvement lacked a statistical correlation with clinical outcomes [9], and was supported by a few other studies [10]. However, larger studies have criticized these changes, highlighting EPE as a significant prognostic factor for pancreatic cancer and arguing that the new AJCC 8th Edition T staging system does not accurately reflect prognosis [11, 12].

Therefore, this study aims to investigate whether imaging findings differ according to presence or absence of EPE in s-PDA, and to identify significant imaging and clinicopathologic features related to recurrence after surgery.

## Materials and methods

This retrospective study was approved by the Institutional Review Board (IRB No. 2311-038-1483), which waived the requirements for informed consent.

### Patients

After reviewing the institution’s databases from January 2004 to October 2021, 150 patients diagnosed with s-PDA measuring 2 cm or less following surgical resection were identified, and their initial CT or MR images were obtained. Among these, three cases were excluded because they were not evaluated using a contrast-enhanced pancreas protocol CT or MRI. Four cases who underwent pancreaticoduodenectomy or hepatojejunostomy for other malignancies prior to s-PDA surgery were excluded. And one case that the tumor size was reported “unmeasurable” in the pathology report was also excluded.

**Fig. 1:**
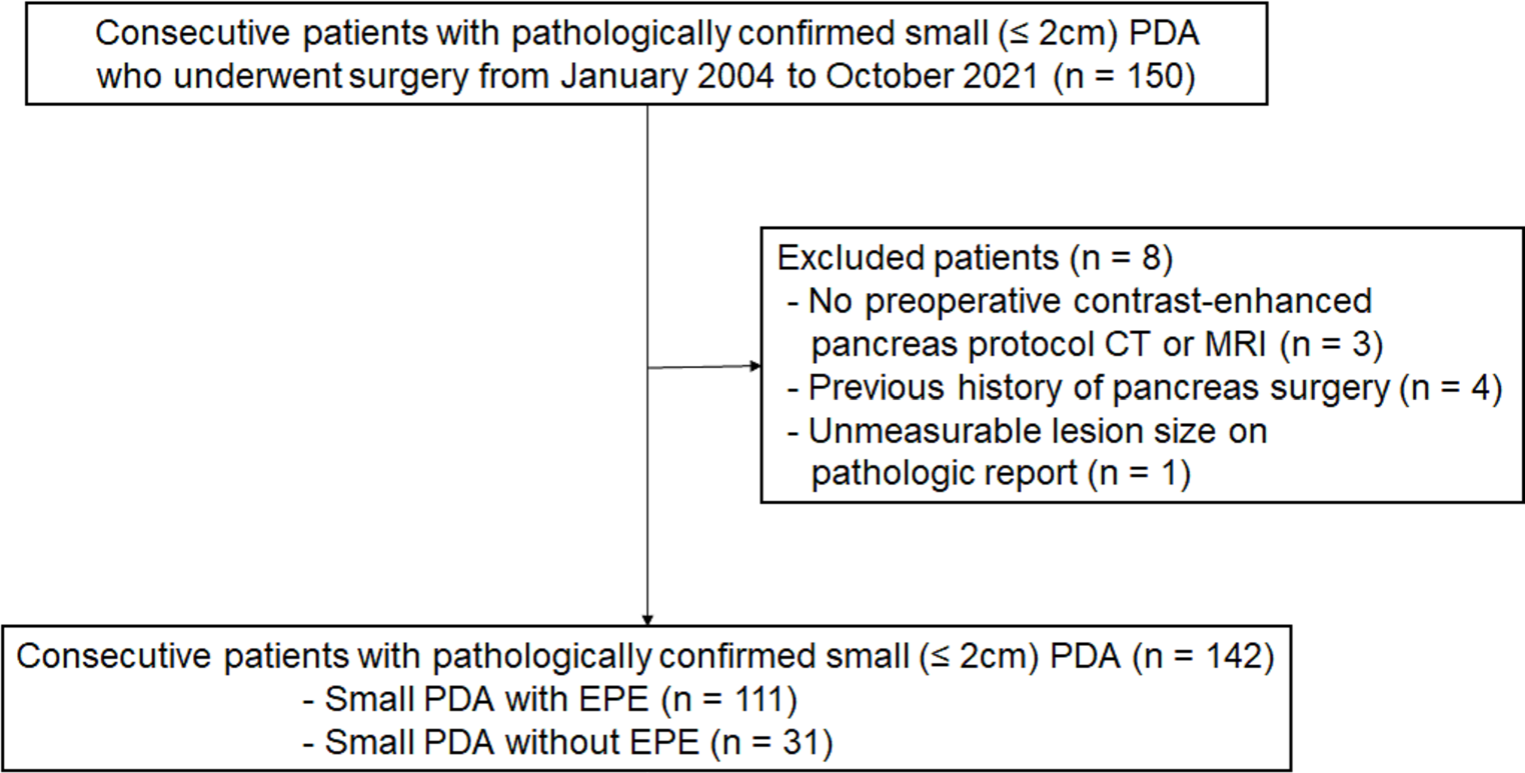
Flow diagram of the included patients

Ultimately, a total of 142 patients (77 male and 65 female; mean age, 64.7 ± 9.3 years) were included in this study (Fig. 1). All patients had a tumor size of 2 cm or less, corresponding to the AJCC 8th Edition pathological T1 stage. The surgeries performed were pancreaticoduodenectomy (*n* = 78, 54.9%), distal pancreatectomy (*n* = 62, 43.7%), and total pancreatectomy (*n* = 2, 1.4%).

### CT examination

Of 142 patients, 134 underwent preoperative CT using commercially available scanners (Supplementary Table 1). Of these, 115 underwent dual-phase MDCT (pre-contrast, pancreatic, and venous phases), and 19 underwent triple-phase multi-detector CT (MDCT) (pre-contrast, pancreatic, venous, and delayed phases). Each patient received 120 mL of non-ionic contrast material at a rate of 2–5 mL/s using an automatic injector. Early arterial phase imaging was delayed by 6 s after reaching 100 Hounsfield unit in the descending aorta, with early and late arterial images acquired during separate breath-holds (5–9 s between scans). Portal venous phase images were obtained 70 s after triggering. CT parameters were as follows: detector configurations, 0.6–0.75 mm; matrix size, 512 × 512; tube voltage, 90–120 kVp; tube current, 120–200 mAs; and rotation time, 0.50–0.75 s. Axial images were reconstructed using slice thickness of 2.5–3 mm, and coronal reconstructions were obtained at a slice thickness of 3 mm using 3D imaging software.

### MRI examination

Of 142 patients, 115 underwent preoperative MRI on 1.5T or 3.0T machines (Supplementary Table 1). The sequences included breath-hold axial T2-weighted imaging (single-shot fast spin echo or half-Fourier acquisition single-shot turbo spin echo), T1-weighted in- and opposed-phase spoiled 3D gradient-echo, DWI using a single-shot echo-planar imaging pulse sequence with b values of 0, 400, and 800 s/mm^2^ (respiratory triggering), and breath-hold T1-weighted fat-suppressed gradient echo. Dynamic imaging used a fat-suppressed 3D gradient echo sequence before and after administering 7.5 mL of Gadovist (1.0 M gadobutrol; Bayer Healthcare, Berlin, Germany) at a 0.1 mmol/kg body weight and a rate of 2 mL/s, followed by a 20–30 mL saline flush. Dynamic phase acquisition timing was monitored using real-time MRI fluoroscopy with placement of the region of interest in the descending aorta. Arterial phase images were obtained 8 s after contrast arrival, and portal venous phase images were obtained at 60 s. Additional delayed phase images were acquired at 2, 3, and 5 min post-injection. The 3D gradient echo data for each phase were collected in a single breath-hold at expiration (mean time, 18 s [range, 16–22 s]).

### Imaging analysis

Two abdominal radiologists, J.H.P. and S.J.H., each with 5 years of experience, independently reviewed anonymized and randomly distributed images. They were blinded to clinical information and radiologic reports except for tumor location. They evaluated preoperative CT and MR images for tumor size, diameter of the main pancreatic duct (MPD) and CBD, relationship between the tumor and major vessels: celiac axis, common hepatic artery (CHA), superior mesenteric artery (SMA), main portal vein (MPV), and superior mesenteric vein (SMV); presence of extrapancreatic neural invasion (EPNI), metastatic lymph nodes (LNs), and resectability. Tumor size was measured in the most clearly visible phase of dynamic imaging; measurements were omitted if evaluation was not possible due to iso-attenuation or intensity. The largest MPD and CBD diameters were measured regardless of phase and plane. Duct dilatation was defined as greater than 3 mm for the upstream MPD [13] and 7 mm or more for the CBD [14]. Tumor-vessel relationships were categorized as no involvement, abutment, or encasement. The tumor-vessel relationships were classified as follows: no involvement when the tumor does not contact the vessel, abutment when the tumor is in direct contact with the vessel but the contact is less than 180° of the vessel’s diameter, and encasement when the tumor contacts more than 180° of the vessel’s diameter or causes a change in caliber, contour irregularity, or occlusion [15]. EPNI was assessed based on five major pathways: from the celiac ganglia to the posterior surface of the pancreatic head (plexus pancreaticus capitalis 1); from the bilateral celiac ganglion to the left margin of the uncinate process via the plexus and around the SMA (plexus pancreaticus capitalis 2); from the plexus around the CHA to the pancreatic head’s anterior region coursing along the gastroduodenal artery; from the left celiac ganglia to the pancreatic body and tail via the splenic artery plexus; and from the left celiac ganglia and plexus to the posterior region of the pancreatic body [16–18]. EPNI was scored on a 3-point scale, as follows: 1, normal pancreatic fat tissue; 2, streaky or strand-like infiltrations in fat tissue observed along known EPNI pathways, regardless of their continuity to the tumor; 3, irregular masses adjacent to tumor along known EPNI pathways, with or without direct continuity to the tumor. Scores of 2 and 3 indicated EPNI. Metastatic LNs were defined as necrotic or exceeding 10 mm in short-axis diameter. Resectability was assessed based on the most widely used and relatively recently revised NCCN guidelines (Supplementary Table 2), taking into account the tumor’s location. Discrepancies were resolved by a third reviewer (J.H.K., with 24 years of experience in abdominal imaging).

### Pathology reference standard

All resected specimens were stained with hematoxylin and eosin staining. The entire portion of the resected specimen examined under a light microscope by two board-certified pathologists in our institution. The gross and microscopic descriptions of the specimens provided in the pathological reports were reviewed retrospectively. The pathological reports included data regarding the diagnosis, differentiation, location of tumor, size of tumor, depth of invasion (including presence of EPE), presence of venous invasion (including large vessels), presence of perineural invasion, tumor (T) and nodal (N) status according to AJCC 8th Edition staging system, and resection status according to the International Union Against Cancer. Resection status was assessed as no residual tumor (R0), microscopic residual tumor (R1), or macroscopic residual tumor (R2).

### Recurrence assessment

All patients underwent postoperative follow-up with CT or MRI every 3–6 months, either alone or alternately. Surveillance intervals were modified based on patient’s risk factors and other considerations. Recurrence was defined as a new enhancing soft tissue lesion in the remnant pancreas or resection margin, or the presence of metastatic lesions on contrast-enhanced CT, MRI, or PET-CT. Recurrence-free survival (RFS) was defined as the interval from surgery to recurrence or, if there was no recurrence, to the last follow-up image.

### Statistical analysis

Clinicopathological and imaging findings were compared between s-PDA with and without EPE using the independent t-test and Mann-Whitney U test for continuous data, and Pearson’s chi-squared and Fischer’s exact tests for categorical data. Variables with *p* <.05 were included in stepwise multivariable analysis. Univariate and multivariate Cox regression analyses were used to identify significant predictors of recurrence. We calculated the optimism-adjusted area under the ROC curve (AUC) of significant predictors. Inter-observer agreement for imaging findings was calculated using the intraclass correlation coefficient (ICC) for continuous variables and κ statistics for categorical variables, interpreted as follows: poor (< 0.20); fair (0.21–0.40); moderate (0.41– 0.60); substantial (0.61–0.80); and near-perfect (0.81–1.00) [19, 20]. Statistical analyses were performed using SPSS software (version 22; SPSS Inc.) and R statistical software (version 4.2.0; R Foundation for Statistical Computing).

## Results

Of 142 patients, 21.8% (*n* = 31) had s-PDA without EPE, while 78.2% (*n* = 111) had s-PDA with EPE. The mean pathological tumor size was 16.5 ± 3.8 mm, significantly larger in s-PDA with EPE **(**13.4 ± 4.7 mm vs. 17.4 ± 3.0 mm; *p* <.001). Tumor differentiation, the presence of metastatic LNs, and recurrence rates (32.3% [10/31] vs. 53.2% [59/111]; *p* =.04) also significantly differed according to EPE. Clinicopathological features of the patients are summarized in Table 1.

**Table 1:** Clinical and histopathologic characteristics of patients with small PDA according to EPE

	Total (*n* = 142)	s-PDA without EPE (*n* = 31)	s-PDA with EPE (*n* = 111)	*P*
Age (years)	64.7 ± 9.3	64.2 ± 9.5	64.8 ± 9.2	0.76
Sex				0.37
Male	77 (54.2%)	19 (61.3%)	58 (52.3%)	
Female	65 (45.8%)	12 (38.7%)	53 (47.7%)	
CA19-9 (U/mL)	1699.7 ± 14942.9	137.8 ± 352.1	2135.8 ± 16890.9	0.23
Tumor location				0.10
Head/uncinate process	74 (52.1%)	11 (35.5%)	63 (56.8%)	
Body/tail	68 (47.9%)	20 (64.5%)	48 (43.2%)	
Operation				0.19
Pancreaticoduodenectomy	78 (54.9%)	13 (41.9%)	65 (58.6%)	
Distal pancreatectomy	62 (43.7%)	17 (54.8%)	45 (40.5%)	
Total pancreatectomy	2 (1.4%)	1 (3.2%)	1 (0.9%)	
R status				0.13
R0	127 (89.4%)	30 (96.8%)	97 (87.4%)	
R1 or R2	15 (10.6%)	1 (3.2%)	14 (12.6%)	
Pathologic tumor size (mm)	16.5 ± 3.8	13.4 ± 4.7	17.4 ± 3.0	< 0.001
Tumor differentiation				0.004
WD	22 (15.5%)	10 (32.3%)	12 (10.8%)	
MD	104 (73.2%)	17 (54.8%)	87 (78.4%)	
PD	16 (11.3%)	4 (12.9%)	12 (10.8%)	
Pathologic N staging (AJCC 7th )				0.002
N0	96 (67.6%)	28 (90.3%)	68 (61.3%)	
N1	46 (32.4%)	3 (9.7%)	43 (38.7%)	
Pathologic N staging (AJCC 8th )				0.009
N0	96 (67.6%)	28 (90.3%)	68 (61.3%)	
N1	40 (28.2%)	3 (9.7%)	37 (33.3%)	
N2	6 (4.2%)	0 (0%)	6 (5.4%)	
Recurrence				0.04
Recur (-)	73 (51.4%)	21 (67.7%)	52 (46.8%)	
Recur (+)	69 (48.6%)	10 (32.3%)	59 (53.2%)	

Values are given as number, mean ± standard deviation

PDA, pancreatic ductal adenocarcinoma; EPE, extrapancreatic extension; CA19-9, carbohydrate antigen 19-9; WD, well differentiated; MD, moderate differentiated; PD, poorly differentiated

### Imaging findings of s-PDA according to EPE

Imaging findings of s-PDA according to EPE are summarized in Table 2. Mean tumor size was 17.4 ± 7.2 mm on CT and 17.2 ± 7.1 mm on MRI. Both CT and MRI measured larger than the pathological size (16.5 ± 3.8 mm), without a statistically significant difference (CT, *p* =.17; MRI, *p* =.20).

**Table 2:** Imaging findings of patients with small PDA according to EPE

	CT				MRI			
	Total (*n* = 134)	s-PDA without EPE (*n* = 29)	s-PDA with EPE (*n* = 105)	*P*	Total (*n* = 115)	s-PDA without EPE (*n* = 29)	s-PDA with EPE (*n* = 86)	*P*
Size (mm)	17.4 ± 7.2	14.3 ± 8.7	18.2 ± 6.5	0.01	17.2 ± 7.1	15.7 ± 7.8	17.7 ± 6.8	0.62
MPD dilatation				0.16				0.10
Absent	57 (42.5%)	9 (31.0%)	48 (45.7%)		38 (33.0%)	6 (20.7%)	32 (37.2%)	
Present	77 (57.5%)	20 (69.0%)	57 (54.3%)		77 (67.0%)	23 (79.3%)	54 (62.8%)	
CBD dilatation				0.24				0.63
Absent	75 (56.0%)	19 (65.5%)	56 (53.3%)		52 (45.2%)	12 (41.4%)	40 (46.5%)	
Present	59 (44.0%)	10 (34.5%)	49 (46.7%)		63 (54.8%)	17 (58.6%)	46 (53.5%)	
Vessel relationship								
CA				-				-
No involvement	134 (100%)	29 (100%)	105 (100%)		115 (100%)	29 (100%)	86 (100%)	
Abutment	0 (0%)	0 (0%)	0 (0%)		0 (0%)	0 (0%)	0 (0%)	
Encasement	0 (0%)	0 (0%)	0 (0%)		0 (0%)	0 (0%)	0 (0%)	
CHA				1.00				-
No involvement	133 (99.3%)	29 (100%)	104 (99.0%)		115 (100%)	29 (100%)	86 (100%)	
Abutment	1 (0.7%)	0 (0%)	1 (1.0%)		0 (0%)	0 (0%)	0 (0%)	
Encasement	0 (0%)	0 (0%)	0 (0%)		0 (0%)	0 (0%)	0 (0%)	
SMA				1.00				1.00
No involvement	131 (97.8%)	29 (100%)	102 (97.1%)		112 (97.4%)	28 (96.6%)	84 (97.7%)	
Abutment	3 (2.2%)	0 (0%)	3 (2.9%)		3 (2.6%)	1 (3.4%)	2 (2.3%)	
Encasement	0 (0%)	0 (0%)	0 (0%)		0 (0%)	0 (0%)	0 (0%)	
MPV				0.87				0.54
No involvement	121 (90.3%)	26 (89.7%)	95 (90.5%)		106 (92.2%)	28 (96.6%)	78 (90.7%)	
Abutment	9 (6.7%)	2 (6.9%)	7 (6.7%)		7 (6.1%)	1 (3.4%)	6 (7.0%)	
Encasement	4 (3.0%)	1 (3.4%)	3 (2.8%)		2 (1.7%)	0 (0%)	2 (2.3%)	
SMV				0.02				0.51
No involvement	87 (64.9%)	25 (86.2%)	62 (59.1%)		79 (68.7%)	22 (75.9%)	57 (66.3%)	
Abutment	40 (29.9%)	3 (10.4%)	37 (35.2%)		34 (29.6%)	7 (24.1%)	27 (31.4%)	
Encasement	7 (5.2%)	1 (3.4%)	6 (5.7%)		2 (1.7%)	0 (0%)	2 (2.3%)	
EPNI				0.24				1.00
Absent	117 (87.3%)	27 (93.1%)	90 (85.7%)		109 (94.8%)	28 (96.6%)	81 (94.2%)	
Present	17 (12.7%)	2 (6.9%)	15 (14.3%)		6 (5.2%)	1 (3.4%)	5 (5.8%)	
Metastatic lymph node				0.64				0.55
Absent	120 (89.6%)	26 (89.7%)	94 (89.5%)		99 (86.1%)	24 (82.8%)	75 (87.2%)	
Present	14 (10.4%)	3 (10.3%)	11 (10.5%)		16 (13.9%)	5 (17.2%)	11 (12.8%)	
Resectability				0.15				0.68
Resectable	120 (89.6%)	28 (96.6%)	92 (87.6%)		108 (93.9%)	28 (96.6%)	80 (93.0%)	
Borderline	14 (10.4%)	1 (3.4%)	13 (12.4%)		7 (6.1%)	1 (3.4%)	6 (7.0%)	
Unresectable	0 (0%)	0 (0%)	0 (0%)		0 (0%)	0 (0%)	0 (0%)	

Values are given as number, mean ± standard deviation

MPD, Main pancreatic duct; CBD, Common bile duct; CA, Celiac axis; CHA, Common hepatic artery; SMA, superior mesenteric artery; MPV, main portal vein; SMV, superior mesenteric vein; EPNI, extrapancreatic neural invasion

**Fig. 2:**
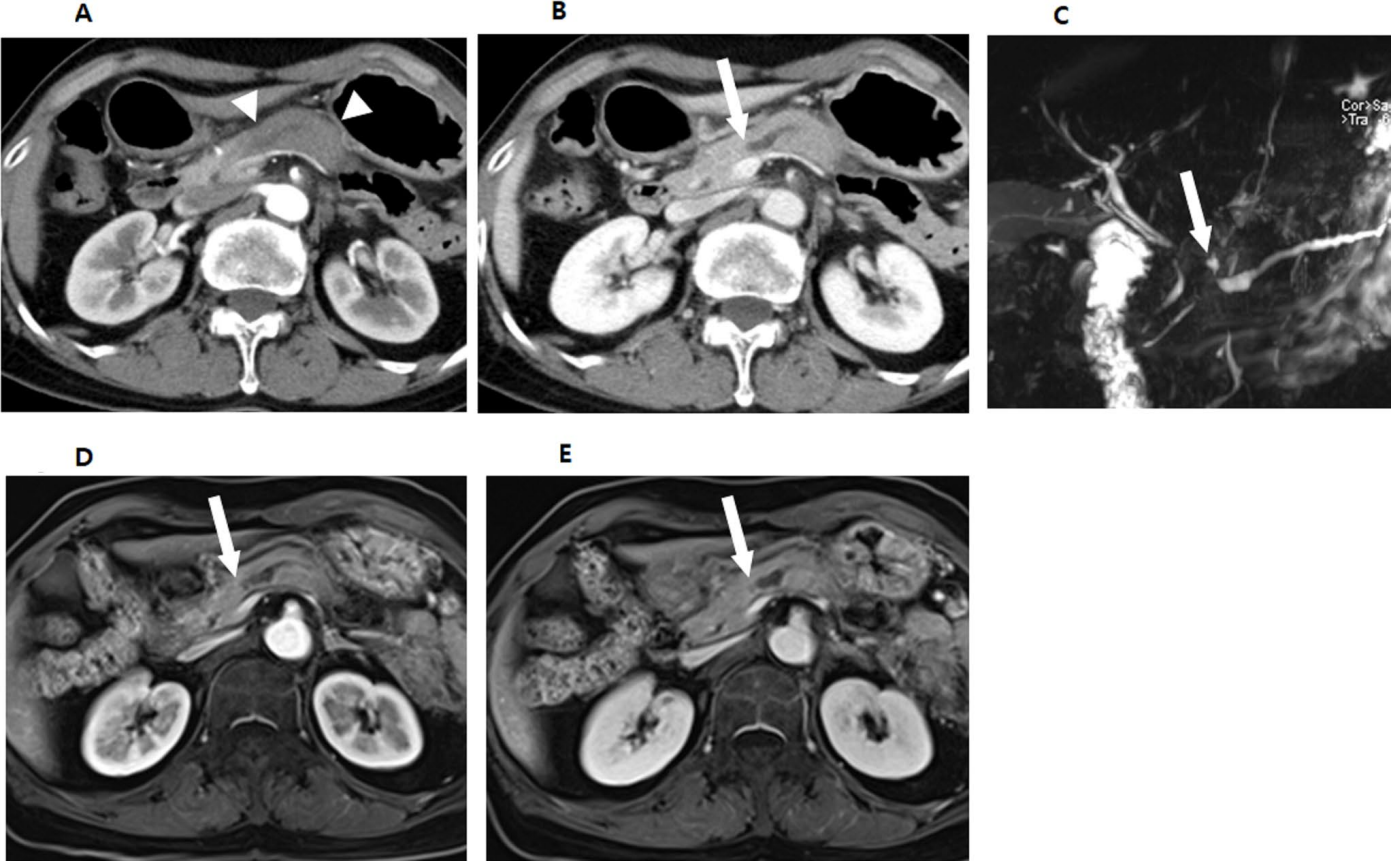
A 66-year-old female patient with small pancreatic adenocarcinoma. **A**,** B.** Contrast-enhanced axial arterial and portal phase CT images show a cutoff and dilatation of main pancreatic duct (arrow) in body portion with combined pancreatic swelling (arrowheads). **C**. MR cholangiography image also shows a stricture of main pancreatic duct with a tiny cyst (arrow) in pancreas body. **D**,** E.** Contrast-enhanced axial T1-weighted arterial and portal phase MR images also show a stricture of main pancreatic duct (arrow) in body portion without definite mass. She underwent a pylorus-preserving pancreaticoduodenectomy and was confirmed histologically to have an 8 mm pancreatic adenocarcinoma without extrapancreatic extension.

**Fig. 3:**
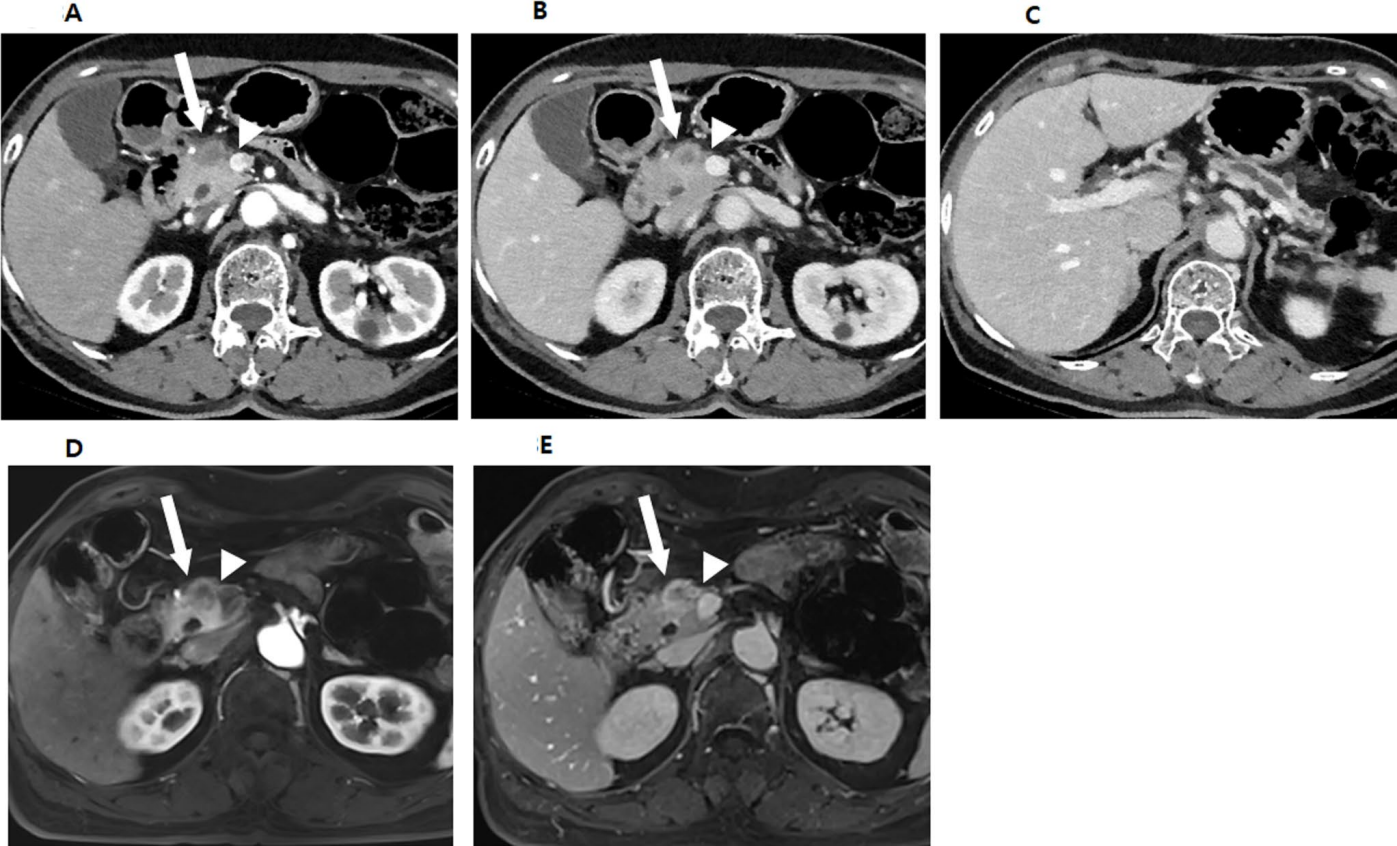
A 75-year-old female patient diagnosed with small pancreatic adenocarcinoma with extrapancreatic extension. **A-C.** Contrast-enhanced axial arterial and portal phase CT images show a hypovascular mass in pancreatic head (arrows). The tumor abuts SMV (arrowheads) and causes dilatation of upstream main pancreatic duct. **D**,** E.** Contrast-enhanced axial T1-weighted arterial and portal MR images also show a hypovascular mass in pancreatic head (arrows) with abutment of the SMV (arrowheads). She was deemed borderline resectable due to SMV abutment and underwent a pylorus-preserving pancreaticoduodenectomy. Histologically, she was diagnosed with a 15 mm pancreatic adenocarcinoma with confirmed extrapancreatic extension, but no SMV invasion. And the surgical margin was not involved by the tumor (R0).

On CT, 8.2% (11/134) and 4.5% (6/134) of tumors were isoattenuating and, thus, invisible to the two reviewers. MPD or CBD dilatation was noted in 75.4% (101/134) of patients (Fig. 2), with 57.5% (77/134) exhibiting MPD dilatation and 44.0% (59/134) exhibiting CBD dilatation. Abutment or encasement of MPV or SMV occurred in 38.1% (51/134) and of the major arteries in only 3.0% (4/134). Interobserver agreements for CT findings ranged from fair to substantial, except for the relationship of CHA (Supplementary Table 3). Tumor size on CT was significantly larger in s-PDA with EPE (18.2 ± 6.5 mm) compared to without EPE (14.3 ± 8.7 mm) (*p* =.01). Abutment or encasement of SMV was more common in s-PDA with EPE (40.9% [43/105]) compared to without EPE (13.8% [4/29]; *p* =.02) (Fig. 3). Tumor size on CT for predicting EPE showed a sensitivity of 81.0%, specificity of 51.7%, and AUC of 0.654 (95% CI, 0.567–0.734; *P* =.02). The Abutment or encasement of SMV for predicting EPE showed a sensitivity of 41.0%, and specificity of 86.2%.

On MRI, 7.8% (9/115) and 6.1% (7/115) of cases were unmeasurable due to iso-intensity assessed by the two reviewers. MPD or CBD dilatation was found in 82.6% (95/115) of patients (Fig. 2); 67.0% (77/115) had MPD dilatation and 63.5% (73/115) had CBD dilatation. Abutment or encasement of the MPV or SMV was present in 36.5% (42/115) and major arteries in only 2.6% (3/115) of patients. EPNI was observed in 8.7% (10/115) of patients. Interobserver agreement for MRI ranged from fair to near-perfect (Supplementary Table 3). Tumor size on MRI was larger in s-PDA with EPE (17.7 ± 6.8 mm) than without EPE (15.7 ± 7.8 mm); however, the difference was not statistically significant (*p* =.62). In our study, EPNI was identified in 12.7% (17/134) of patients on CT and 8.7% (10/115) on MRI, which was considerably lower than the 59.2% (84/142) observed in the pathological reports.

### Clinicopathological and imaging findings for predicting tumor recurrence

**Fig. 4:**
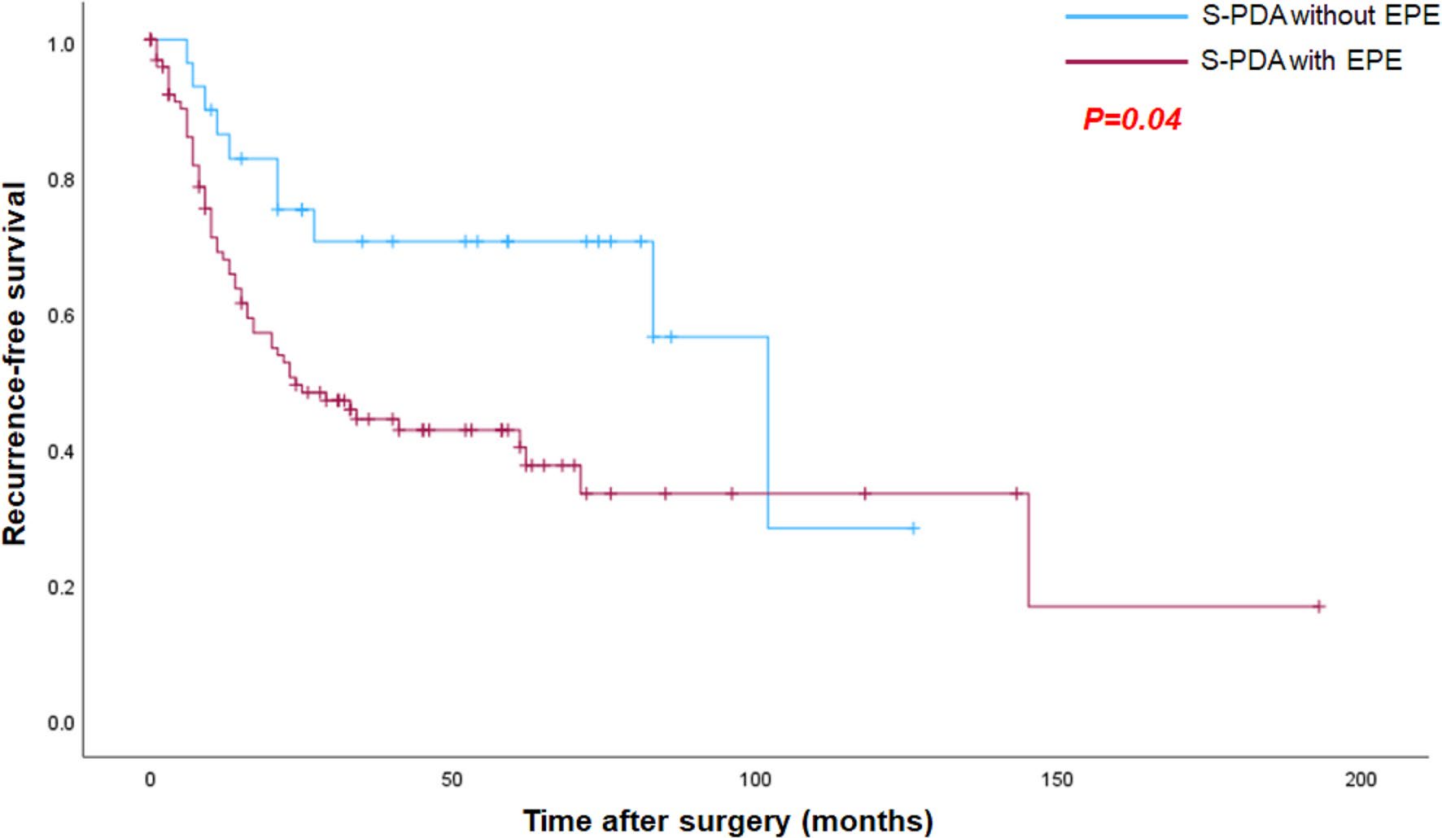
Kaplan-Meier curve comparing recurrence-free survival between the Small PDA without and with EPE

**Fig. 5:**
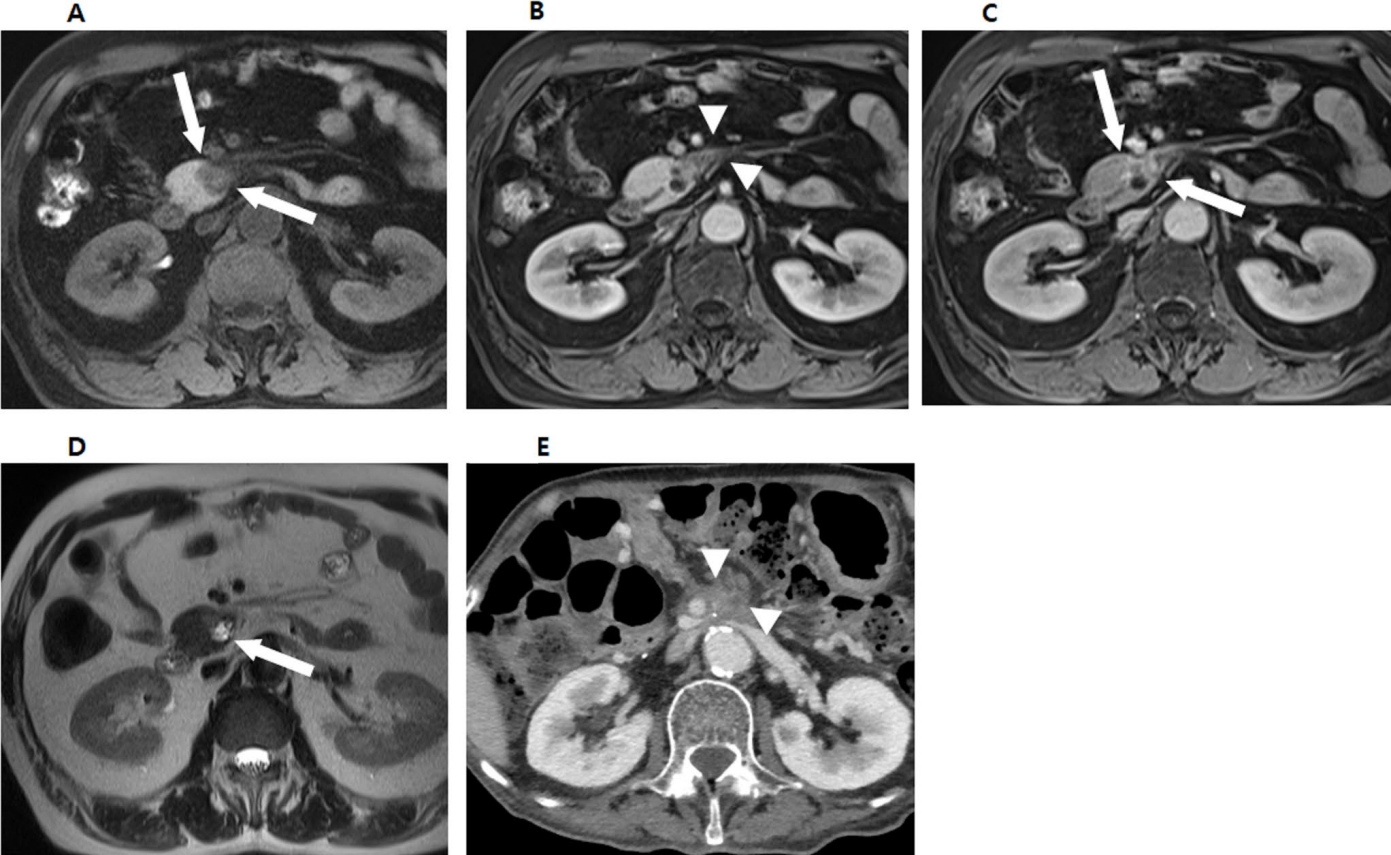
A 65-year-old male patient with recurrence after small pancreatic adenocarcinoma surgery. **A**-**C**. Axial T1-weighted MR image and contrast-enhanced axial T1-weighted arterial, and portal phase MR images show a hypovascular mass in pancreatic uncinate process (arrows). Irregular mass-like infiltrations (arrowheads) are noted adjacent to the tumor. **D.** Axial T2-weighted MR image shows a combined cyst in pancreatic uncinate process (arrow). He underwent pylorus-preserving pancreaticoduodenectomy and histologically confirmed a 1.5 cm pancreatic adenocarcinoma with perineural invasion. There was no large vessel invasion, including SMV, and the surgical margin was free from tumor. **E.** 13 months after surgery, tumor recurrence (arrowheads) was detected at the operation site on follow-up contrast-enhanced axial portal phase CT image.

Recurrence occurred in 48.6% (69/142) of patients, with a median RFS of 61.0 months (95% confidence interval [CI] 21.2–100.8 months). For patients with s-PDA without EPE, the recurrence rate was 32.3% (10/31), with a median RFS of 102 months. In contrast, s-PDA with EPE had a higher recurrence rate (53.2% [59/111]) and a significantly shorter median RFS of 24 months (*p* =.04) (Fig. 4). Significant clinicopathological predictors of recurrence included serum CA19-9 level (*p* =.02), pathological tumor size (*p* =.004), tumor differentiation (*p* =.03), and the presence of EPE (*p* =.01). In the multivariate Cox regression analysis, only pathological tumor size (HR 1.103; 95% CI: 1.020–1.193; *p* =.01) was a significant predictor of recurrence. Among the CT findings, only the vessel relationship of the SMV was significant in the univariate Cox regression analysis (*p* =.04). Among the MRI findings, tumor size (*p* =.02), the vessel relationship of the SMA (*p* =.01), and the presence of EPNI (*p* <.001) were significant predictors of recurrence. Multivariate Cox regression analysis revealed that tumor size (HR 1.044; 95% CI: 1.001–1.090, *p* =.048) and presence of EPNI (HR 3.341; 95% CI: 1.564–7.140, *p* =.002) were significant predictors (Fig. 5). The tumor size on MRI for predicting recurrence showed a sensitivity of 79.6%, specificity of 33.3%, AUC of 0.563 (95% CI, 0.467–0.656; *P* =.24). The presence of EPNI for predicting recurrence showed a sensitivity of 16.7%, and specificity of 98.4%. Clinicopathological and imaging features that predicted recurrence are summarized in Tables 3 and 4.

**Table 3:** Important clinical and histopathologic findings for prediction of tumor recurrence in patients with small PDA

	Univariate cox regression			Multivariate cox regression		
	HR	95% CI	*P*	HR	95% CI	*P*
Age (years)	0.992	0.966–1.018	0.55			
Sex						
Male						
Female	0.811	0.504–1.305	0.39			
CA19-9	1.312	1.051–1.639	0.02	-	-	-
Tumor location						
Head/uncinate						
Body/tail	0.739	0.458–1.194	0.22			
R status						
R0						
R1 or R2	0.852	0.368–1.972	0.71			
Pathologic tumor size (mm)	1.120	1.038–1.209	0.004	1.103	1.020–1.193	0.01
Tumor differentiation			0.03			0.08
WD						
MD	2.778	1.195–6.460	0.02	2.225	0.948–5.220	0.07
PD	1.266	0.357–4.490	0.72	1.062	0.298–3.789	0.93
Pathologic T staging (AJCC 7th )						
T1						
T3	2.318	1.182–4.549	0.01	-	-	-
Pathologic N staging (AJCC 7th )						
N0						
N1	1.224	0.745–2.012	0.43			
Pathologic N staging (AJCC 8th )			0.27			
N0						
N1	1.100	0.643–1.882	0.73			
N2	2.232	0.846–5.888	0.11			

Values are given as number, mean ± standard deviation

CA19-9, carbohydrate antigen 19-9; WD, well differentiated; MD, moderate differentiated; PD, poorly differentiated

**Table 4:** Important imaging findings for prediction of tumor recurrence in patients with small PDA

	CT						MRI					
	Univariate cox regression			Multivariate cox regression			Univariate cox regression			Multivariate cox regression		
	HR	95% CI	*P*	HR	95% CI	*P*	HR	95% CI	*P*	HR	95% CI	*P*
Size (mm)	1.023	0.988–1.059	0.21				1.049	1.007–1.093	0.02	1.044	1.001–1.090	0.048
MPD dilatation												
Absent												
Present	0.932	0.571–1.520	0.78				1.189	0.655–2.158	0.57			
CBD dilatation												
Absent												
Present	1.525	0.935–2.489	0.09				1.709	0.970–3.012	0.06			
Vessel relationship												
CA												
No involvement												
Abutment												
Encasement	-	-	-				-	-	-			
CHA												
No involvement												
Abutment												
Encasement	0.003	0.00–425E + 096	0.26				-	-	-			
SMA										-	-	-
No involvement												
Abutment												
Encasement	2.512	0.79–7.987	0.17				7.160	2.116–24.224	0.01			
MPV												
No involvement												
Abutment												
Encasement	1.292	0.737–2.265	0.40				1.410	0.709–2.802	0.37			
SMV												
No involvement												
Abutment												
Encasement	1.550	1.037–2.316	0.04				1.479	0.910–2.405	0.13			
EPNI												
Absent												
Present	1.114	0.531–2.335	0.78				4.073	1.999–8.302	< 0.001	3.341	1.564–7.140	0.002
Metastatic LN												
Absent												
Present	0.694	0.297–1.625	0.40				1.830	0.962–3.480	0.07			
Resectability												
Resectable												
Borderline	1.964	0.999–3.861	0.050				1.993	0.787–5.049	0.15			
Unresectable												

Values are given as number, mean ± standard deviation

MPD, Main pancreatic duct; CBD, Common bile duct; CA, Celiac axis; CHA, Common hepatic artery; SMA, superior mesenteric artery; MPV, main portal vein; SMV, superior mesenteric vein; EPNI, extrapancreatic neural invasion; LN, lymph node

## Discussion

Even in s-PDA, EPE is common. Among 142 patients in our study, 78.2% (*n* = 111) had EPE. s-PDA with EPE had larger pathologic tumor size (13.4 ± 4.7 mm vs. 17.4 ± 3.0 mm, *p* <.001) and a higher recurrence rate (32.3% [10/31] vs. 53.2% [59/111]; *p* =.04). The common CT findings predicting EPE in s-PDA included larger tumor size (14.3 ± 8.7 mm vs. 18.2 ± 6.5 mm; *p* =.01) and abutment or encasement of the SMV (13.8% [4/29] vs. 40.9% [43/105]; *p* =.02). MPD or CBD dilatation were common imaging findings of s-PDA (CT, 75.4% [101/134]; MRI, 82.6% [95/115]) and imaging tended to measure the size larger compared to pathological size, although the difference was not statistically significant. On MRI, tumor size and EPNI were significant predictors of recurrence.

EPE is an important prognostic factor for pancreatic cancer. In a study involving 1506 patients with T1 PDA from 43 centers, those with EPE exhibited shorter median survival (39 vs. 107 months; *p* <.001) [11]. Another study also reported a shorter median survival for T1 PDA with EPE (24 vs. 43 months; *p* <.001) [25]. Similarly, our study found that tumors with EPE were common in s-PDA (78.2% [111/142]), had significantly larger pathological tumor sizes compared to those without (*p* <.001), and shorter median RFS (*p* =.04). Tumor size on CT (*p* =.01) and abutment or encasement of the SMV on CT (*p* =.02) were also significantly different according to the presence of EPE. These results emphasize that EPE remains an important prognostic factor and suggest that reintegrating EPE into pancreatic cancer staging may improve prognostic accuracy and, moreover, that it is important to predict the presence or absence of EPE through imaging before surgery.

Tumor size is also a well-known prognostic factor for PDA [21]. The AJCC 8th Edition classifies tumors as T1–T3 based on size, with T1 further subcategorized [22]. Previous studies have shown that tumors measured on CT and MRI tend to be smaller than those measured using pathological techniques [22–25]. Possible reasons include tumor shrinkage during pathological processing, the time gap between imaging and surgery, or measurement variability due to the lack of standardized orientation. However, in our study, the tumor sizes measured on CT and MRI were larger than the pathological sizes. The reasons are difficult to ascertain without case-by-case comparison, but possible factors include that a PDA typically presents as an ill-defined hypoattenuating mass due to fibrous stroma, which can lead radiologists to overestimate its size, especially since pancreatic cancer is often overestimated on imaging as well. Unlike the majority of PDA, which are observed as hypoattenuating masses due to fibrous stroma and decreased vascularity [26], s-PDA appears more commonly as an isoattenuating mass [5]. Rupert et al. and Frank et al. reported that 11% and 5.4–14% of PDAs were iso-attenuated on CT, respectively [6, 27]. In such cases, direct diagnosis of the primary tumor may be difficult; therefore, identifying secondary signs is important [4]. In a study by Yoon, 76% of the s-PDAs, including 88% of the isoattenuating tumors, were accompanied by secondary signs [5]. Our study revealed that 8.2% (11/134) of isoattenuating tumors on CT and 7.8% (9/115) of iso-intense tumors on MRI, with similar rates across modalities. The lower incidence compared with that in previous studies may be attributed to secondary signs being common (81.8% [9/11] on CT and 88.9% [8/9] on MRI). These results are consistent with those reported by other studies [4, 6, 27] and emphasize the need for careful evaluation of secondary signs, especially in s-PDA.

In our study, EPNI was identified in 12.7% (17/134) of patients on CT and 8.7% (10/115) on MRI, which was considerably lower than the 59.2% (84/142) observed in the pathological reports. This suggests that EPNI observed on imaging does not always perfectly correspond to true tumor infiltration. In a retrospective study involving 76 patients with PDA, Chang et al., demonstrated that hypoatteuating tumor infiltrating vessels could be used to diagnose EPNI [28]. In a study involving 384 patients with PDA, Guo et al. found that a minimum distance of 6.5 mm between the tumor and CA or SMA yielded a sensitivity of 71.6% and a specificity of 84.3% for diagnosing EPNI [29]. Taken together, EPNI remains a challenging imaging finding, difficult to differentiate. Furthermore, its detection is influenced by factors such as the experience of the radiologist and image quality. However, in our study, EPNI detected on MRI was a significant predictor of recurrence in s-PDA (HR 3.341, 95% CI 1.564–7.140, *p* =.002). Several other studies have also reported that EPNI is a significant prognostic factor in pancreatic cancer [16, 30, 31]. Therefore, further research on EPNI is necessary, and careful evaluation by radiologists is essential.

The present study had several limitations, the first of which was its retrospective design, which may have introduced selection bias. In particular, because we only included patients diagnosed with s-PDA, the number of cases with vascular abutment or encasement was small; therefore, the tumor-vessel relationship was evaluated by categorizing it as ‘contact’ or ‘no contact’. As such, it did not reflect the entire s-PDA spectrum. Second, this study utilized the pathological report as a reference through a retrospective review. It was not feasible to compare CT or MR images with pathology for every case. A prospective study would be necessary to systematically compare imaging with pathology. Third, because preoperative CT and MRI were performed using different devices, different image qualities may have affected the evaluation of imaging findings. However, this heterogeneity may reflect actual, real-world clinical practice. Lastly, while thin (preferably submillimeter) slice-thickness CT is recommended for the optimal assessment of pancreatic cancer [15], this study utilized CT with a thicker slice thickness of 2.5–3 mm. As a result, there may be limitations in the evaluation of tumor-vessel relationship and EPNI, and further studies using higher-quality pancreatic CT would be desirable.

Even in s-PDA (≤ 2 cm), tumors with EPE tend to be larger and exhibit higher recurrence rates. While there are differences in clinicopathological findings depending on the presence or absence of EPE, imaging features play a crucial role in predicting the presence of EPE.

## Electronic supplementary material

Below is the link to the electronic supplementary material.

Supplementary Material 1

## Data Availability

Data availability statement: The datasets generated or analyzed during the study are available from the corresponding author on reasonable request.

## Abbreviations

AJCC: American Joint Committee on Cancer
CBD: Common Bile Duct
CHA: Common Hepatic Artery
EPE: ExtraPancreatic Extension
EPNI: ExtraPancreatic Neural Invasion
LN: Lymph Node
MPD: Main Pancreatic Duct
MPV: Main Portal Vein
NCCN: National Comprehensive Cancer Network
PDA: Pancreatic Ductal Adenocarcinoma
SMA: Superior Mesenteric Artery
SMV: Superior Mesenteric Vein
