# Supplementary material for: Small pancreatic ductal adenocarcinoma (≤ 2 cm): different imaging and clinicopathologic features according to extrapancreatic extension
Source: Abdom Radiol (NY). 2025 Feb 14;50(9):4168–80. doi: 10.1007/s00261-025-04831-0 (PMC12331785; doi:10.1007/s00261-025-04831-0)
Supplement: Supplementary file 1 — Supplementary Material 1 [file 261_2025_4831_MOESM1_ESM.docx]

**Supplement materials**

**Supplementary Table 1.** Types of machines used in this study.

| **CT** | **N** | **MR** | **N** |
| --- | --- | --- | --- |
| Brilliance 64 (Philips Healthcare) | 28 | Verio (Siemens Medical Solutions) | 25 |
| SOMATOM Definition (Siemens Healthcare) | 17 | Skyra (Siemens Medical Solutions) | 23 |
| Dual-source systems IQon (Philips Healthcare) | 16 | Ingenia (Philips Healthcare) | 17 |
| Sensation 16 (Siemens Healthcare) | 14 | Achieva (Philips Healthcare) | 15 |
| SOMATOM Force (Siemens Healthcare) | 13 | Signa (Philips Healthcare) | 9 |
| Billiance iCT (Philips Healthcare) | 12 | Biograph_mMR (Siemens Medical Solutions) | 7 |
| Ingenuity (Philips Healthcare) | 9 | TrioTim (Siemens Medical Solutions) | 4 |
| LightSpeed (GE Healthcare) | 9 | Ananto (Siemens Medical Solutions) | 4 |
| Aquilion ONE (Toshiba Medical Systems) | 6 | Discovery MR750 (Philips Healthcare) | 3 |
| Discovery CT 750 HD (GE Healthcare) | 5 | Sonata (Philips Healthcare) | 3 |
| Sensation 64 (Siemens Healthcare) | 3 | Magnetom Vida (Siemens Medical Solutions) | 2 |
| MX8000 (Marconi Medical Systems) | 2 | Intera (Philips Healthcare) | 2 |
|  |  | Espree (Siemens Medical Solutions) | 1 |

**Supplementary Table 2.** Criteria defining resectability status at diagnosis according to National Comprehensive Cancer Network Guideline (Version 1.2021).

| **Resectability Status** | **Arterial** | **Venous** |
| --- | --- | --- |
| Resectable | No arterial tumor contact (CA, SMA, or CHA) | No tumor contact with the SMV or PV or ≤ 180° contact without vein contour irregularity |
| Borderline  Resectable | Pancreatic head/uncinated process: | Solid tumor contact with the SMV or PV of > 180°, contact of ≤ 180° with contour irregularity of the vein or thrombosis of the vein but with suitable vessel proximal and distal to the site of involvement allowing for safe and complete resection and vein reconstruction  Solid tumor contact with the IVC |
|  | Solid tumor contact with CHA without extension to CA or hepatic artery bifurcation allowing for safe and complete resection and reconstruction |  |
|  | Solid tumor contact with SMA of ≤180° |  |
|  | Solid tumor contact with variant arterial anatomy (ex: accessory right hepatic artery, replaced right hepatic artery, replaced CHA, and the origin of replaced or accessory artery) and the presence and degree of tumor contact should be noted if present, as it may affect surgical planning |  |
|  | Pancreatic body/tail: |  |
|  | Solid tumor contact with the CA of ≤ 180° |  |
|  | Solid tumor contact with the CA of > 180° without involvement of the aorta and with intact and uninvolved GDA thereby permitting a modified Appleby procedure (some panel members prefer these criteria to be in the locally advanced category) |  |
| Unresectable | Head/uncinated process: | Unreconstructible SMV/PV due to tumor involvement or occlusion (can be due to tumor or bland thrombus) |
|  | Solid tumor contact with SMA or CA > 180° |  |
|  | Pancreatic body/tail: |  |
|  | Solid tumor contact of > 180° with the SMA or CA |  |
|  | Solid tumor contact with the CA and aortic involvement |  |

**Supplementary Table 3.** Summary of the inter-reader agreement for each imaging findings.

|  | **CT** | **MR** |
| --- | --- | --- |
|  | Interobserver agreement (95% CI) | Interobserver agreement (95% CI) |
| Size (mm) | 0.517 (0.381 – 0.631) | 0.832 (0.765 – 0.880) |
| MPD dilatation | 0.62 (0.49 – 0.75) | 0.58 (0.42 – 0.75) |
| CBD dilatation | 0.58 (0.45 – 0.71) | 0.33 (0.16 – 0.50) |
| Vessel relationship |  |  |
| CA | - | - |
| CHA | -0.0075 (-0.018 – -0.0029) | - |
| SMA | 0.49 (0.055 – 0.92) | 0.74 (0.40 – 1.00) |
| MPV | 0.66 (0.44 – 0.88) | 0.40 (0.095 – 0.70) |
| SMV | 0.52 (0.37 – 0.68) | 0.44 (0.26 – 0.62) |
| EPNI | 0.35 (0.16 – 0.54) | 0.37 (0.1 – 0.64) |
| Metastatic LN | 0.41 (0.14 – 0.68) | 0.27 (0.012 – 0.52) |
| Resectability | 0.36 (0.099 – 0.62) | 0.38 (0.12 – 0.64) |
